# 944. CMV Peak Viral Load, Recurrence, Duration, and Outcomes in Kidney Transplant Recipients

**DOI:** 10.1093/ofid/ofab466.1139

**Published:** 2021-12-04

**Authors:** Robin K Avery, Robin K Avery, Darin B Ostrander, Na Lu, Felicia Akinwande, Min Young Kim, Shilpa Gopinath, Nitipong Permpalung, Obichukwu Ezennia, Yuexin Tang, Kieren Marr

**Affiliations:** 1 Johns Hopkins, Baltimore, MD; 2 Johns Hopkins University, Baltimore, Maryland; 3 Seoul Medical Center, Seoul, Seoul-t’ukpyolsi, Republic of Korea; 4 Johns Hopkins School of Medicine, Baltimore, Maryland; 5 Johns Hopkins University School of Medicine, Baltimore, MD; 6 Merck and Co., Inc, North wales, Pennsylvania; 7 John Hopkins, Bethesda, Maryland

## Abstract

**Background:**

Cytomegalovirus (CMV) infection continues to cause morbidity in kidney transplant recipients, despite prophylaxis and pre-emptive therapy. Predictors of poor outcomes remain incompletely characterized. We questioned whether markers of CMV replication (CMV peak viral load, recurrent episodes, or duration of CMV DNAemia) are associated with adverse outcomes in the current era.

**Methods:**

We studied 605 people who underwent kidney transplant at Johns Hopkins University (2010 – 2018). Mean follow-up was 45.5 months. The average age was 51.85 years and 39.7% were female. Donor-seropositive, recipient seronegative (D+/R-) patients received valganciclovir 900 mg/day for 6 months, while R+ patients received valganciclovir 450 mg/day for 3 months. CMV recurrence was defined as CMV DNAemia after two undetectable CMV PCR’s. Outcomes of acute rejection, graft failure, and death were evaluated in univariate analysis; p values were calculated by Fisher’s exact test.

**Results:**

Peak CMV viral load was not associated with any outcomes (Table 1). There was a trend of increased graft failure in people who had long duration ( >6 month) DNAemia (Table 2). More than two episodes of CMV reactivation was associated with graft failure and rejection (Table 3).

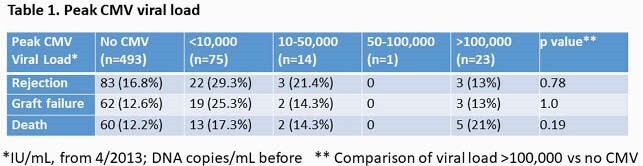

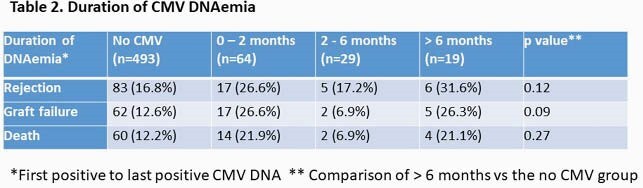

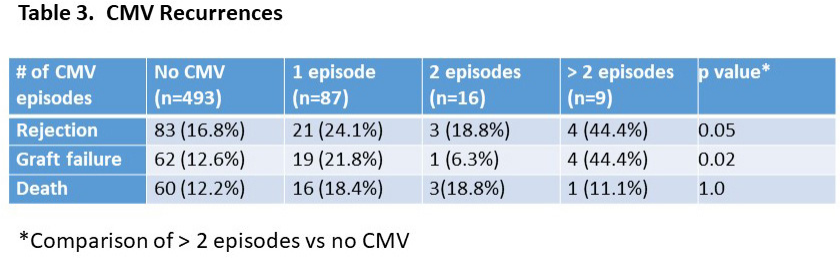

**Conclusion:**

CMV reactivation is associated with kidney rejection and failure in univariate models. Multivariate analyses and longitudinal modeling will provide increased data upon which to better instruct preventative strategies.

**Acknowledgments:**

Funding for the research study was provided by Merck Sharp & Dohme Corp., a subsidiary of Merck & Co., Inc., Kenilworth, NJ, USA

**Disclosures:**

**Robin K. Avery, MD**, **Aicuris** (Grant/Research Support)**Astellas** (Grant/Research Support)**Chimerix** (Research Grant or Support)**Merck** (Grant/Research Support)**Oxford Immunotec** (Grant/Research Support)**Qiagen** (Grant/Research Support)**Takeda/Shire** (Grant/Research Support) **Yuexin Tang, PhD**, **JnJ** (Other Financial or Material Support, Spouse’s employment)**Merck & Co., Inc.** (Employee, Shareholder) **Kieren Marr, MD**, **Merck** (Grant/Research Support, Advisor or Review Panel member)

